# Viral Immune signatures from cerebrospinal fluid extracellular vesicles and particles in HAM and other chronic neurological diseases

**DOI:** 10.3389/fimmu.2023.1235791

**Published:** 2023-08-09

**Authors:** Michelle L. Pleet, Joshua A. Welsh, Emily H. Stack, Sean Cook, Dove-Anna Johnson, Bryce Killingsworth, Tim Traynor, Annaliese Clauze, Randall Hughes, Maria Chiara Monaco, Nyater Ngouth, Joan Ohayon, Yoshimi Enose-Akahata, Avindra Nath, Irene Cortese, Daniel S. Reich, Jennifer C. Jones, Steven Jacobson

**Affiliations:** ^1^ Viral Immunology Section, Neuroimmunology Branch, National Institute of Neurological Disorders and Stroke, National Institutes of Health, Bethesda, MD, United States; ^2^ Translational Nanobiology Section, Laboratory of Pathology, Center for Cancer Research, National Cancer Institute, National Institutes of Health, Bethesda, MD, United States; ^3^ Section of Infections of the Nervous System, National Institute of Neurological Disorders and Stroke, National Institutes of Health, Bethesda, MD, United States; ^4^ Experimental Immunotherapeutics Unit, National Institute of Neurological Disorders and Stroke, National Institutes of Health, Bethesda, MD, United States; ^5^ Translational Neuroradiology Section, National Institute of Neurological Disorders and Stroke, National Institutes of Health, Bethesda, MD, United States

**Keywords:** HTLV-1, HAM, extracellular vesicles, CSF, T-cells, viral infection, neurological disease

## Abstract

**Background and objectives:**

Extracellular vesicles and particles (EVPs) are released from virtually all cell types, and may package many inflammatory factors and, in the case of infection, viral components. As such, EVPs can play not only a direct role in the development and progression of disease but can also be used as biomarkers. Here, we characterized immune signatures of EVPs from the cerebrospinal fluid (CSF) of individuals with HTLV-1-associated myelopathy (HAM), other chronic neurologic diseases, and healthy volunteers (HVs) to determine potential indicators of viral involvement and mechanisms of disease.

**Methods:**

We analyzed the EVPs from the CSF of HVs, individuals with HAM, HTLV-1-infected asymptomatic carriers (ACs), and from patients with a variety of chronic neurologic diseases of both known viral and non-viral etiologies to investigate the surface repertoires of CSF EVPs during disease.

**Results:**

Significant increases in CD8+ and CD2+ EVPs were found in HAM patient CSF samples compared to other clinical groups (*p* = 0.0002 and *p* = 0.0003 compared to HVs, respectively, and *p* = 0.001 and *p* = 0.0228 compared to MS, respectively), consistent with the immunopathologically-mediated disease associated with CD8+ T-cells in the central nervous system (CNS) of HAM patients. Furthermore, CD8+ (*p* < 0.0001), CD2+ (*p* < 0.0001), CD44+ (*p* = 0.0176), and CD40+ (*p* = 0.0413) EVP signals were significantly increased in the CSF from individuals with viral infections compared to those without.

**Discussion:**

These data suggest that CD8+ and CD2+ CSF EVPs may be important as: 1) potential biomarkers and indicators of disease pathways for viral-mediated neurological diseases, particularly HAM, and 2) as possible meditators of the disease process in infected individuals.

## Introduction

Human T-lymphotropic virus type 1 (HTLV-1), the first human retrovirus to be discovered, results in a chronic, lifelong infection (1). While most people infected with HTLV-1 remain permanent asymptomatic carriers (ACs), up to 5% can develop adult T-cell leukemia/lymphoma (ATLL), an aggressive T-cell cancer that can be located in the lymph nodes, blood, skin, or other areas of the body. Additionally, up to 4% of infected individuals develop the chronic, neuroinflammatory myelopathy called HTLV-1-associated myelopathy (HAM), which shares many clinical similarities to some forms of another chronic progressive demyelinating disease, multiple sclerosis (MS) (2–4). During pathogenesis, HTLV-1-infected CD4+ T-cells in the central nervous system (CNS) induce an inflammatory positive feedback loop that ultimately results in CNS damage. Furthermore, infected cells generate several viral products, such as the HTLV-1 Tax protein. Tax normally acts as the activator of viral transcription; however, Tax can also drive the activation and expansion of Tax-specific CD8+ T-cells (5). While these CD8+ cytotoxic T-lymphocytes (CTLs) play a critical role in controlling the proviral load in HTLV-1-infected individuals (6), it has been suggested that they are immunopathogenic since HAM patients have substantially higher CTL responses and elevated proviral loads compared to ACs (7, 8). In HAM patients, Tax-specific CTLs are chronically activated, indicative of continuous exposure to Tax protein (9).

Chronic neurologic disease encompasses a wide spectrum of etiologies including genetic, autoimmune, protein disorders, and infection-driven pathologies (10). Differentiating and diagnosing of many of these diseases can be challenging, since neurologic presentations are often nonspecific with attributes shared between multiple diseases. The diagnostic challenges are further compounded by the lack of clear etiological agents in their respective development (11). For example, many groups over the years have proposed viral infection as a possible trigger for MS, although as of yet no clear consensus has been reached (12). A bourgeoning avenue of exploration in the fields of human health and disease are extracellular vesicles (EVs). Extracellular vesicles and particles (EVPs) are routinely released from virtually all cell types as part of normal cell biology; however, during disease and infection, EVPs are known to often package inflammatory elements including proteins, RNAs, and lipids, which can thereby functionally affect recipient cells (13, 14). Indeed, EVPs have been explored in many studies of viral infection and have been shown to package viral components (i.e. proteins or RNAs) or even in some cases entire virions, which can then aid in the cell-to-cell spread of infection or cause direct damage to recipient cells (15–17). EVPs can play not only a direct role in the development and progression of disease, but they can also be used as biomarkers. Previously, our group has demonstrated that HTLV-1 Tax protein can be packaged into small, membrane-bound EVs which are released from HTLV-1 infected cells, and can thereby sensitize recipient cells for an HTLV-1-specific CTL response (18). We hypothesized that by sampling the EVPs from the cerebrospinal fluid (CSF) of patients with HAM and other neurological diseases, these compartmentalized EVPs may contain information relating to their cellular origin and may thereby implicate pathogenic mechanisms at work. Here, we analyzed the EVPs from the CSF of healthy volunteers (HVs) and patients with a variety of chronic neurologic diseases of both known viral and non-viral etiologies including HAM, HTLV-1 ACs, MS, and other neurologic diseases (ONDs) for 39 surface membrane markers against various immunological, structural, and EV-associated targets.

## Materials and methods

### Ethics statement

CSF samples used in this study were collected from subjects under the National Institute of Neurologic Disorders and Stroke protocols #98-N-0047, 89-N-0045, 13-N-0149, 13-N-0017, and 15-N-0125. Prior to study inclusion, written informed consent was obtained from the subject in accordance with the Declaration of Helsinki.

### Human samples, isolation, and storage

CSF samples were obtained by lumbar puncture in house at the NIH clinical center. HVs enrolled under NINDS protocols were self-reported as healthy and did not require lumbar puncture for any diagnostic or medical reason. Immediately after collection, samples were centrifuged at 1,300 x *g* for 10 minutes. The cell-free supernatants were collected in cryotubes in 1 mL aliquots and immediately frozen at -80°C until use. The collected CSF cells were counted using a Muse cell analyzer (Millipore) and freshly used for immunophenotyping analysis. For this study, the cell-free CSF material was used for our assays. We selected 10 HV, 10 MS, 10 HAM, and 14 OND CSF samples to provide sufficient numbers for this exploratory study (**Table 1**). We included 5 AC CSFs as these samples are quite rare.

**Table 1:** Patient cohort demographics.

		# Patients		Male/Female		Median Age (Range)	
**HV**		10		4/6		54 (21-58)	
**HAM**		10		1/9		56.5 (26-62)	
**AC**		5		1/4		52 (31-58)	
**MS**		10		4/6		51 (24-67)	
**ONDs**		14		6/8		48 (28-66)	
**PML**		3		3/0		50 (39-66)	
**HSV-1 encephalitis**		1		0/1		44	
**HSV-2 meningitis**		1		1/0		46	
**Jamestown Canyon virus**		1		0/1		36	
**HIV-1**		2		1/1		55.5 (54-57)	
**NMDA receptor encephalitis**		2		1/1		30 (28-32)	
**neurosarcoidosis**		1		1/0		51	
**Susac syndrome**		1		0/1		43	
**motor neuron disease**		1		0/1		67	
**LHES**		1		0/1		68	

Healthy volunteers (HVs), HTLV-1-associated myelopathy (HAM), HTLV-1 asymptomatic carriers (ACs), multiple sclerosis (MS), and other neurological disease (OND) patient demographics are shown by age in years and sex. Individual ONDs are listed by diagnosis: PML (progressive multifocal leukoencephalopathy), HSV-1 (herpes simplex virus) encephalitis, HSV-2 meningitis, Jamestown Canyon virus, HIV-1 (human immunodeficiency virus), Anti-N-methyl D-aspartate (NMDA) receptor encephalitis, neurosarcoidosis, Susac syndrome, motor neuron disease, and lymphocytic variant hypereosinophilic syndrome (LHES).

### Immunophenotyping

Immunophenotyping in peripheral blood lymphocyte and CSF lymphocyte populations was examined in each subject. EDTA-treated whole blood or CSF cells were stained with CD3, CD4, CD8, and CD45 (all from BD Biosciences), as previously described (19). All flow cytometric analysis was performed using a LSR II (BD Biosciences). The data were analyzed using FlowJo (v10.8.1).

### Microfluidic resistance pulse sensing

CSF EVP size and concentration was measured using MPRS (Spectradyne nCS1™, Spectradyne LLC, USA). Measurements were accomplished using TS-400 microfluidic cartridges. Neat, cell-free CSF samples were measured immediately after first-time freeze-thaw with the addition of a 240 nm NIST bead spike-in population at a final concentration of 1 x 10^9^/mL. Acquired output files were imported into MATLAB (v2022a, Mathworks Inc.) where the spike-in population was used to calibrate the sample size and concentration across acquisitions. Statistical analysis of summary data was performed using GraphPad Prism (v9.3.1).

### Multiplex assay

Cell-free CSF samples were titrated (250, 150, and 50 µL) for incubation with 10 µL of MACSPlex Exosome Kit (Cat No. 130-108-813, Miltenyi Biotec) antibody-capture bead mixture which contains 39 separate fluorescently barcoded bead populations, each specific to a different cell surface marker, in 1.5 mL low protein binding tubes overnight at room temperature, rotating and protected from light. A 0.2 µm PES filter plate was washed using 150 µL of MACSPlex buffer and cleared using a vacuum manifold. Fifty microliters of MACSPlex buffer were added to each well before incubated EVP-capture bead samples were aliquoted into each of the wells. The EVP-capture bead mixtures were washed using vacuum manifold and immediately resuspended in 200 µL MACSPlex detection antibody solutions (equal parts α-CD81, α-CD9, and α-CD63; three commonly used EV-associated tetraspanins). Wells were reverse pipetted to mix, and plates were left on a shaker for two hours at room temperature and protected from light. Samples were washed three times using the vacuum manifold and immediately resuspended in 200 µL MACSPlex buffer. Each well was then transferred to 96-well U-bottom polypropylene plates. Sandwiched EVPs were then analyzed via flow cytometry and signals were detected for EVPs that co-expressed the capture bead marker as well as one or multiple of the EV-enriched tetraspanins. All data acquired by Multiplex assay (MPA) required the sample to be bound by both a capture bead and an EVP tetraspanin-specific detection antibody, and thus we refer to these markers as EVP-associated.

### Flow cytometry

Beads were triggered using a forward light scatter trigger threshold and optimal gains for each detector (Aurora, Cytek Bioscience, USA) found by performing voltration on 8-peak beads (Cat. 422903, BioLegend, USA). Each plate well was acquired using a medium flow rate (~30 µL/min^-1^) until 5,000 single bead events had been acquired. Post-acquisition .fcs file arbitrary unit scales were calibrated to APC molecules of equivalent soluble fluorochrome (MESF) units using reference standards (APC Quantitative Beads Dried Down CTT, Cat. #626425, Becton Dickinson).

### Data analysis

Flow cytometry files were calibrated to MESF units using FCM_PASS_ software (v3.08, https://nano.ccr.cancer.gov/software/) (20, 21). Conversion of .fcs file arbitrary units to MESF units for these assays was performed for the purpose of being able to directly compare the output numbers between this data set and others to be measured in the future on the same or different flow cytometers. For more information on why and how this was accomplished, please refer to the relevant works by Welsh et al. (20, 22). Multiplex bead gates were drawn using the calibrated data in FlowJo (v10.8.1), exported to .csv files, and imported in MPA_PASS_ software (v1.01, https://nano.ccr.cancer.gov/software/) where a database was built. A detailed protocol can be found at http://dx.doi.org/10.17504/protocols.io.bm3gk8jw (23). A normalized dataset was built by performing background subtraction of unstained control beads from all other samples, resulting the analysis data using MESF units above background. Statistical analyses between multiple disease groups were performed in GraphPad Prism (v9.3.1). Separate control and disease groups were analyzed by Kruskal-Wallis with Dunn’s multiple comparisons tests for significance. Viral vs. non-viral groups were subjected to Mann-Whitney tests for significance.

## Results

### Development of a workflow to analyze CSF EVP signatures

**Figure 1:**
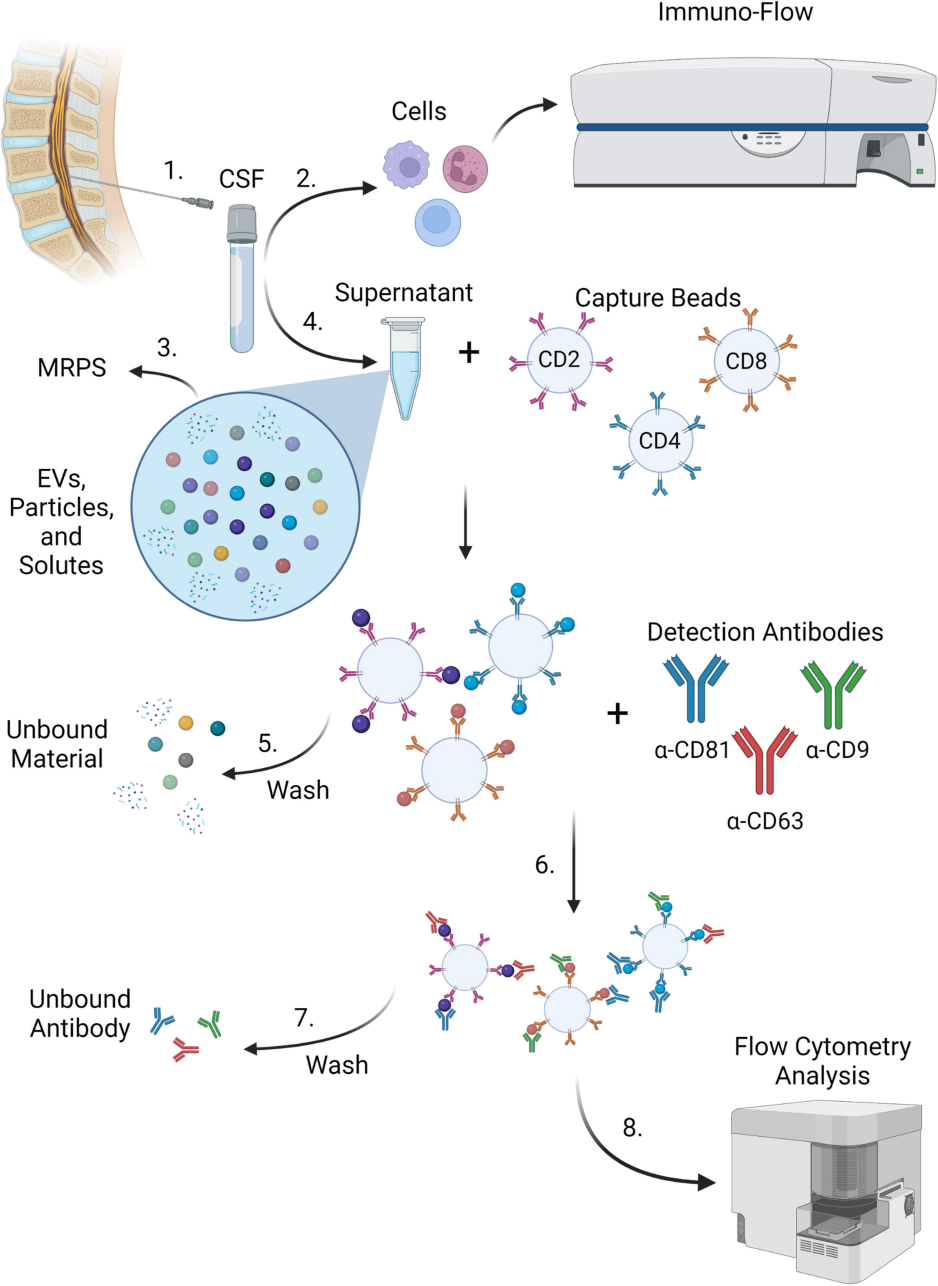
MPA workflow. 1) CSF was acquired from donors via lumbar puncture. 2) Cells were removed from CSF samples and subjected to immuno-flow phenotyping for immune markers. 3) Supernatants containing EVs, extracellular particles, and other solutes were measured by MRPS with the nCS1 particle size analyzer. 4) Cell-free supernatants from CSF containing EVs, extracellular particles, free proteins, and other solutes were incubated with a cocktail of fluorescently barcoded capture beads conjugated to antibodies targeting cell surface epitopes such as CD2, CD4, and CD8. EVPs bound to corresponding capture beads were retained and (5) wash steps were performed to remove unbound EVPs and free proteins. 6) EVP-bound capture beads were then incubated with detection antibodies (such as CD81, CD63, and CD9 targeting EV-enriched surface tetraspanins). Capture bead-EVP-detection antibody complexes were retained and (7) subjected to wash steps in order to remove excess unbound detection antibodies. 8) EVP-bound complexes were then analyzed by flow cytometry. Figure created with BioRender.

Previously, we have studied EVPs from both peripheral blood mononuclear cells (PBMCs) and the CSF of HAM and MS patients and found that EVs from HTLV-1-infected cells can contain Tax protein and were able to sensitize an HTLV-1-specific CTL response in targets (18). Because we had not yet explored the origin of these CSF EVs in depth, we sought to determine whether CSF EVP surface markers differed between HAM, MS, ONDs, and/or HVs. Analysis of EVP surface markers via bead-based capture methods have been optimized on several flow cytometric platforms, including single-EV flow cytometry and fluorescently barcoded multiplex kits (20, 21, 23). Multiplex analysis (MPA) in particular has been a promising avenue for these studies, as it allows one to study individual EVPs based upon multiple customizable capture beads and detection antibodies in a single heterogenous sample. We recently published an optimized analysis pipeline and methodology using MPA kits for a variety of EVP sample types, including from cell culture supernatants, serum, plasma, and CSF samples (23). Here, we utilized these techniques to study CSF EVPs from HVs, patients with HTLV-1 (HAM and ACs), MS, and ONDs. Human samples included in this study are outlined (**Table 1**). Frozen cell-free CSF samples were processed according to the illustrated workflow (**Figure 1**).

### MRPS analysis of EVP concentrations in human CSF

**Figure 2:**
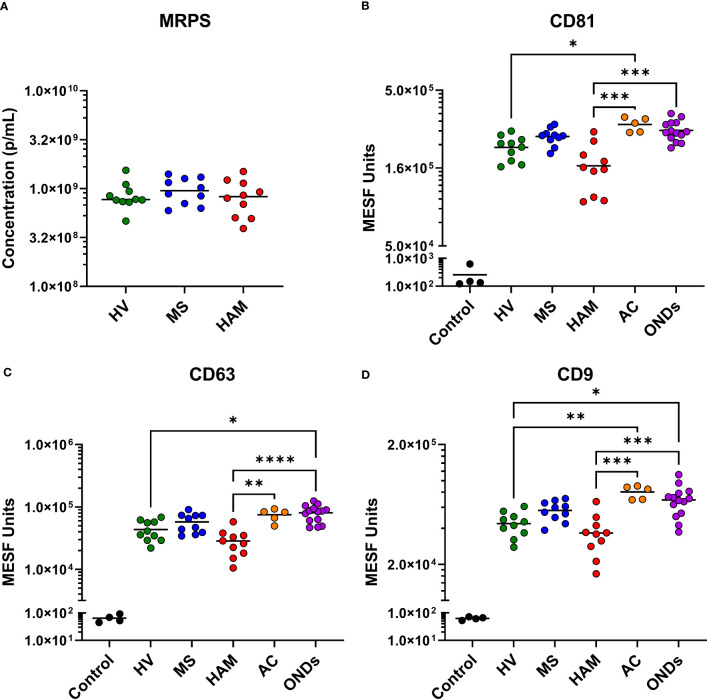
Microfluidic resistive pulse sensing (MRPS) and tetraspanin analysis of CSF EVPs. **(A)** Ten healthy volunteer (HV), multiple sclerosis (MS), and HTLV-1-associated myelopathy (HAM) CSF samples were measured by MRPS (Spectradyne, nCS1). Raw acquisition data was normalized to known NIST standard spike-in beads in MATLAB. Concentrations in particles/mL (p/mL) were graphed comparing each clinical group. Nonparametric statistical analyses comparing groups were performed using Kruskal-Wallis with Dunn’s multiple comparisons tests, showing no statistical differences between groups. MPA results of CSF EVPs from HV, MS, HAM (HAM), AC, and ONDs are shown. Results were normalized and converted from .fcs file arbitrary unit scales to APC MESF units using reference standards and MPA_PASS_ software (https://nano.ccr.cancer.gov/software/) (23). EVPs bound to CD81 **(B)**, CD63 **(C)**, and CD9 **(D)** conjugated capture beads are shown and compared by group. Controls shown are the same capture beads incubated with detection antibodies in the absence of EVP sample, representing the nonspecific background fluorescence of the assay. Nonparametric statistical analyses comparing groups were performed using Kruskal-Wallis with Dunn’s multiple comparisons tests (*p* ≤ 0.05 = *; *p* < 0.01 = **; *p* < 0.001 = ***; *p* < 0.0001 = ****).

Based on our previous work, EVPs are present at such a low concentration in CSF (< 5 x 10^9^ particles/mL) that pre-isolation of EVPs prior to incubation with multiplex beads did not improve EVP attachment or detection with multiplex capture (23); therefore, neat, cell-free CSF was used here. To determine EVP concentration and size distribution, HV, HAM, and MS samples were analyzed by MRPS. Quantification of these analyses (**Figure 2A**) demonstrated that CSF EVPs ≥ 90 nm were present at concentrations ranging from approximately 4 x 10^8^ to 1.5 x 10^9^ particles/mL, in which all groups had similar median concentrations. In all samples tested, the size distribution of EVPs detected was consistent between groups (**Supplementary Figure 1**).

### HAM CSF EVPs have altered surface signatures

Utilizing the developed workflow for the analysis of EVP surface markers (**Figure 1**), we analyzed our cohort of CSF samples by MPA. A heatmap of the normalized data output for all samples, controls, and surface markers is shown (**Supplementary Figure 2**). All samples were run in titration to verify specificity of signals in a dose-dependent manner (**Supplementary Figure 3**). As expected, the EV-enriched tetraspanins CD81, CD9, and CD63 were strongly positive in all CSF samples and across all groups (dark red on left, **Supplementary Figure 2**). CD133/1 and HLA-DR,DP,DQ also presented with strong signals and minor visible variations across groups by heatmap. The most obvious gross changes in signal strength between disease groups appeared to be from EVPs that were CD8+ (red box). Many of the markers included in this MPA that one might expect to be positive in blood (i.e. platelet markers like CD42a, CD41b, and CD62P) were not substantially different from the baseline in these CSF samples.

**Figure 3:**
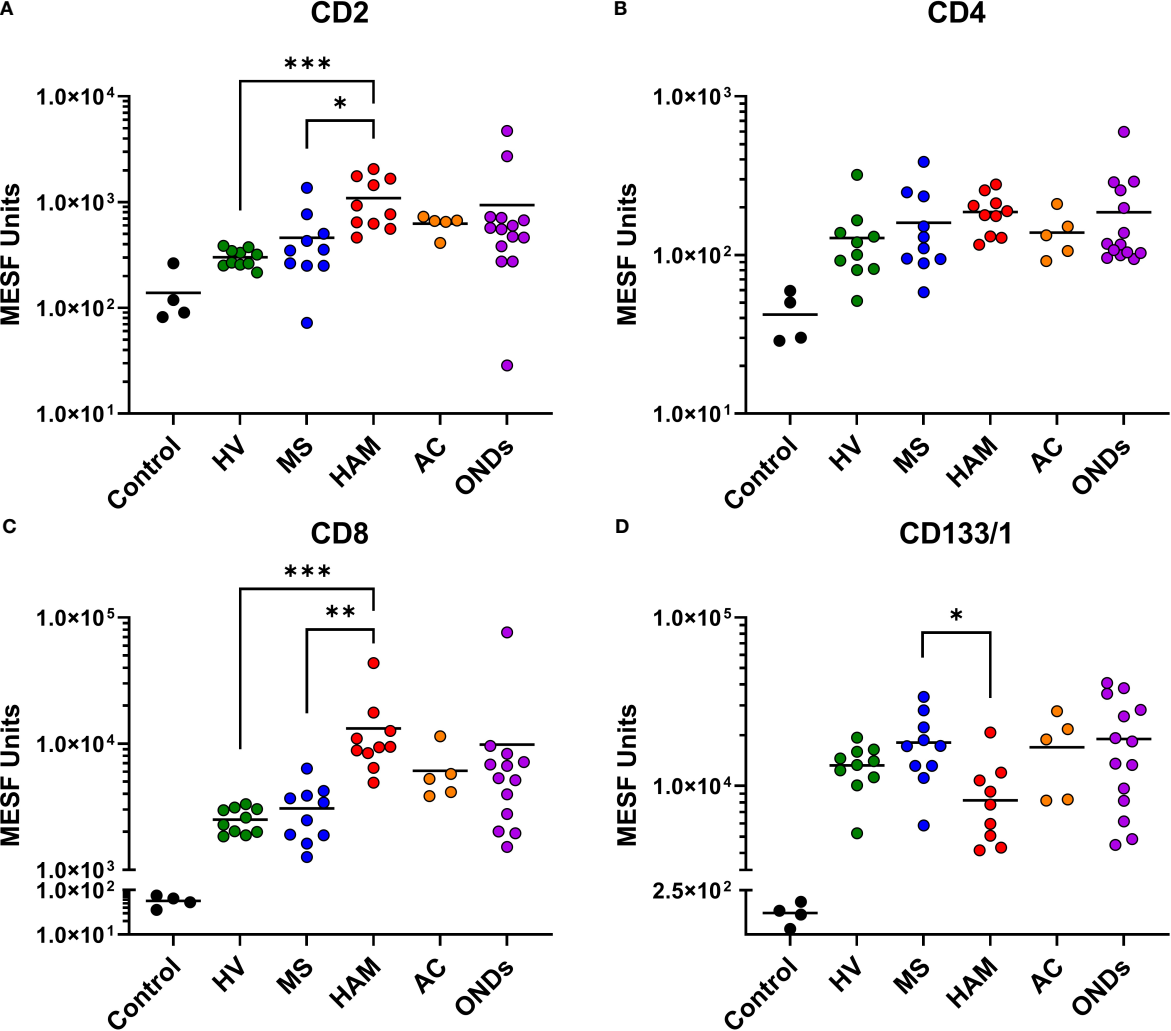
Multiplex analysis (MPA) of CSF EVP surface markers compared by disease group. Selected results of MPA of CSF EVPs from HV, MS, HAM (HAM), AC, and ONDs are shown. Results were normalized and converted from .fcs file arbitrary unit scales to APC MESF units using reference standards and MPA_PASS_ software (https://nano.ccr.cancer.gov/software/) (23). EVPs bound to CD2 **(A)**, CD4 **(B)**, CD8 **(C)**, and CD133/1 **(D)** conjugated capture beads are shown and compared by group. Controls shown are the same capture beads incubated with detection antibodies in the absence of EVP sample, representing the nonspecific background fluorescence of the assay. Nonparametric statistical analyses comparing groups were performed using Kruskal-Wallis with Dunn’s multiple comparisons tests (*p* ≤ 0.05 = *; *p* < 0.01 = **; *p* < 0.001 = ***).

Selected MPA results were converted to MESF units, background subtracted, and compared by clinical group. While all groups had tetraspanin signals > 1 x 10^4^ MESF units, EVPs from the CSF of individuals with HAM showed comparatively decreased levels (**Figures 2B-D**). Specifically, CD81, CD63, and CD9 were decreased in comparison to ACs (*p* = 0.0007, *p* = 0.0068, and *p* = 0.0004, respectively) and ONDs (*p* = 0.0005, *p* < 0.0001, and *p* = 0.006, respectively), but not to HVs and MS patients. Additionally, ACs and ONDs commonly had increased signals of tetraspanins compared to HVs (specifically, ACs: *p* = 0.0316 for CD81 and *p* = 0.0071 for CD9; ONDs: *p* = 0.0128 for CD63 and *p* = 0.0203 for CD9). EVP-associated T-cell markers CD8 and CD2 were increased in HAM patients compared to HVs (*p* = 0.0002 and *p*= 0.0003, respectively) and MS patients (*p* = 0.001 and *p* = 0.0228, respectively), whereas CD4+ EVPs had overall lower signals and were not statistically different between groups (**Figures 3A-C**). HLA-ABC+, HLA-DR,DP,DQ+, CD44+, CD40+, CD14+, and CD24+ EVPs were detectable in all groups (**Supplementary Figure 4**); however, with the exception of HLA-ABC EVPs which had elevated signals in HAM compared to HV (*p* = 0.0085) and CD44+ EVPs which had elevated signals in ACs and ONDs compared to HVs (*p* **=** 0.0175 and *p* = 0.0053, respectively), no other groups differed substantially from each other. Finally, CD133/1+ (a “stemness”-related marker commonly used to detect and isolate cancer stem cells from various solid tumors (24, 25)) EVP signals were decreased in HAM patients compared to other groups, albeit only significantly compared against MS (*p* = 0.0485; **Figure 3D**). All other markers measured and analyzed are shown by disease group (**Supplementary Figure 4**). In order to verify the reproducibility of this data, the experiment was repeated with different HVs, HAM and MS patients and using a new lot of the same MACSPlex Exosome Kit (**Supplementary Figure 5**). CD8+ and CD2+ EVP signals were again significantly increased in the HAM patient CSFs compared to HV (*p* **=** 0.0023 and *p* = 0.0184, respectively) and MS (*p* **=** 0.0002 and *p* = 0.0180, respectively). Additionally, CD133+ EVP signals were reproducibly decreased in HAM compared to MS patient CSFs (*p* = 0.0406). The patient demographics for the second cohort used are shown in **Supplementary Table 1**. Collectively, these data show that EV-enriched tetraspanins, CD8, CD2, and CD133/1, were altered in HAM patient CSF EVPs compared to other disease and control groups.

### CD8+ EVPs in HAM patient CSF correlates with CD8+ T-cells in the CNS

**Figure 4:**
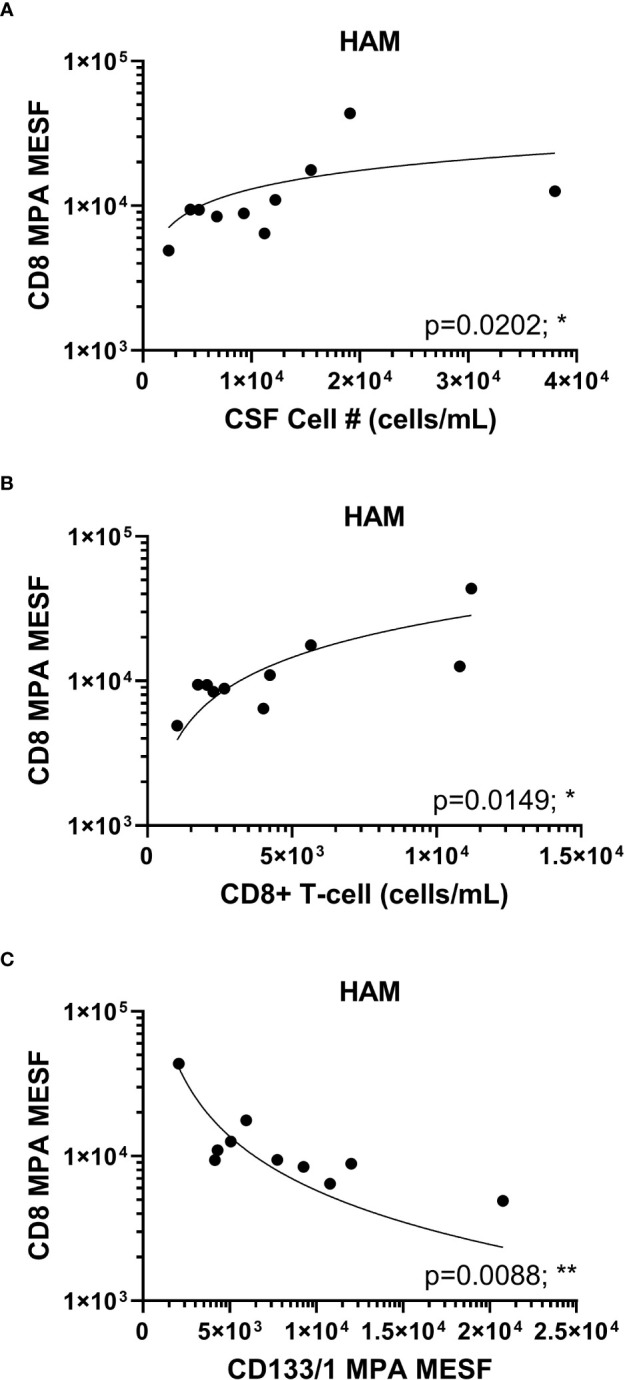
HAM CSF CD8+ EVP correlation plots. CD8+ EVPs (in MESF units) from HAM CSF samples were compared to the concentration of CSF cells **(A)** and CD8+ T-cells **(B)** found in the patient CSF samples by previous immuno-flow analyses. **(C)** CD8+ EVPs (in MESF units) were also compared to the MESF units of CD133/1 from HAM patient CSF. Analyses were performed using Spearman r correlation analysis with 95% confidence intervals (p ≤ 0.05 = *; p < 0.01 = **).

It has been well established that CD8+ T-cells in HAM patients play a critical role in pathogenesis as these cells have been shown to be elevated in the CSF of patients compared to ACs and have been demonstrated in HAM CNS lesions (7, 8, 26). Therefore, the finding that CD8+ EVP signals were elevated in HAM CSF compared to other chronic neurological disease groups and HVs is consistent with the known immunopathology associated with CD8+ T-cells in HAM. We next wished to determine if the CD8+ signal from EVPs in HAM patients were correlated with any other markers or clinical findings. We found CD8+ EVP signals were positively correlated with the total cell (*p* = 0.0202, r = 0.7333) and the CD8+ T-cell concentration (*p* = 0.0149, r = 0.7576) in the CSF of HAM patients (**Figures 4A, B**). In addition, CD8+ EVPs were also strongly negatively correlated (*p* = 0.0088, r = -0.7939) with CD133/1+ EVPs in HAM CSF (**Figure 4C**).

### CSF EVP signatures differ between individuals without viral infection and viral diseases

**Figure 5:**
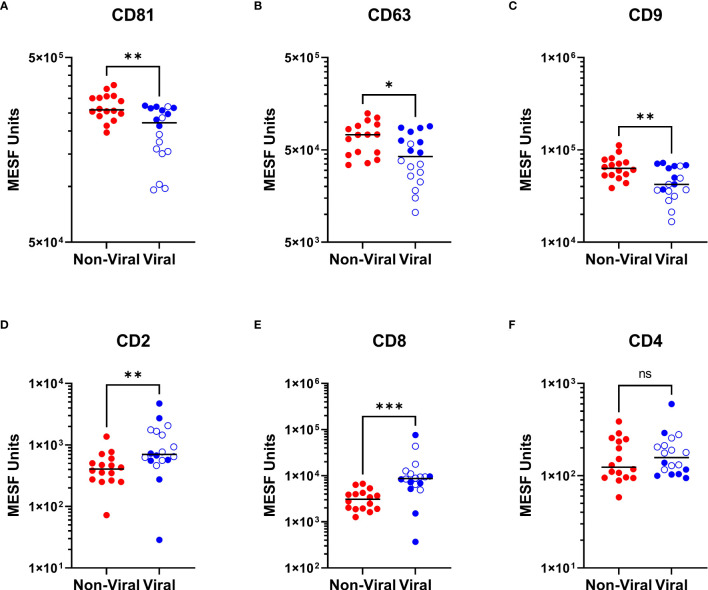
Viral neurologic disease CSF EVP signatures compared to non-viral diseases. MPA results of CSF EVPs from individuals with viral-associated neurologic disease [Viral group: HAM (n=10; open blue circles), PML (n=3), HSV-1 encephalitis (n=1), HSV-2 meningitis (n=1), Jamestown Canyon virus (n=1), and HIV-1 (n=2)] compared to individuals without viral-associated neurological disease [Non-Viral group: MS (n=10), NMDA receptor encephalitis (n=2), neurosarcoidosis (n=1), Susac syndrome (n=1), motor neuron disease (n=1), and LHES (n=1)]. Results were normalized and converted from .fcs file arbitrary unit scales to APC MESF units using reference standards and MPA_PASS_ software. EVPs bound to CD81 **(A)**, CD63 **(B)**, CD9 **(C)**, CD2 **(D)**, CD8 **(E)**, and CD4 **(F)** conjugated capture beads are shown and compared by group. Nonparametric statistical analyses comparing groups were performed using Mann-Whitney tests for significance (ns = not significant; p ≤ 0.05 = *; p < 0.01 = **; p < 0.001 = ***).

**Figure 6:**
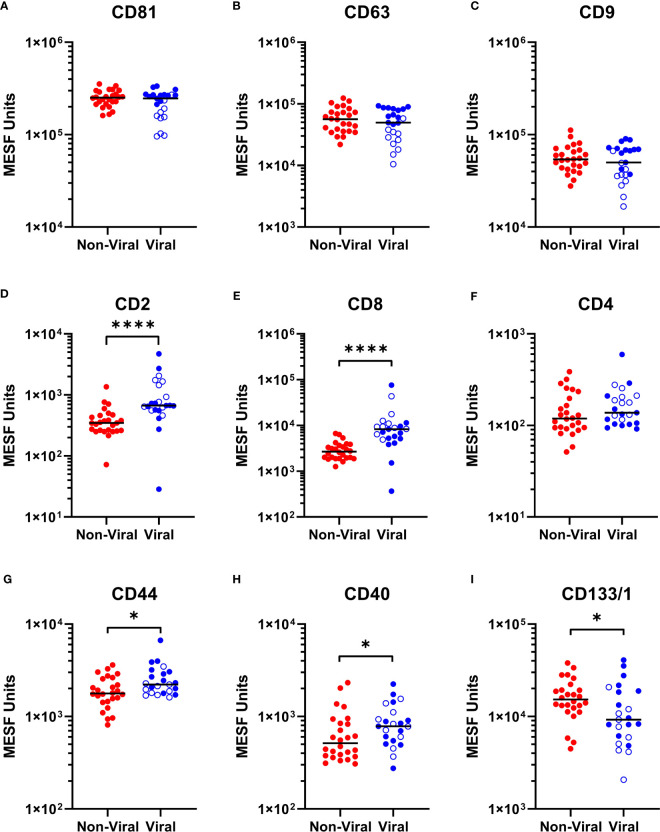
Multiplex analysis (MPA) of CSF EVP surface markers compared by viral vs. non-viral groups. Selected results of MPA of CSF EVPs from viral [HAM (n=10; open blue circles), AC (n=5), PML (n=3), HSV-1 encephalitis (n=1), HSV-2 meningitis (n=1), Jamestown Canyon virus (n=1), and HIV-1 (n=2)] and non-viral [HV (n=10), MS (n=10), NMDA receptor encephalitis (n=2), neurosarcoidosis (n=1), Susac syndrome (n=1), motor neuron disease (n=1), and LHES (n=1)] groups are shown. Results were normalized and converted from .fcs file arbitrary unit scales to APC MESF units using reference standards and MPA_PASS_ software (https://nano.ccr.cancer.gov/software/) (23). EVPs bound to CD81 **(A)**, CD63 **(B)**, CD9 **(C)**, CD2 **(D)**, CD8 **(E)**, CD4 **(F)**, CD44 **(G)**, CD40 **(H)**, and CD133/1 **(I)** conjugated capture beads are shown and compared by group. Open blue circles represent data from HAM CSF EVPs. Nonparametric statistical analyses comparing groups were performed using Mann-Whitney tests for significance (*p* ≤ 0.05 = *; *p* < 0.0001 = ****).

As we were able to observe differences in the relative abundance of EVP surface markers in HTLV-1-infected HAM patients compared to other chronic neurological diseases, particularly in relationship to increased CD8+ EVP signals (**Figure 3C**), we next investigated if there were differences in the CSF EVP signatures in those additional patients associated with viral infections, including PML (JC polyomavirus), HSV-1 encephalitis, HSV-2 meningitis, Jamestown canyon virus, and HIV-1 (**Table 1**). An initial evaluation of viral neurological disease patients compared to CSF from individuals without viral neurological disease was performed. CD81, CD63, and CD9 signals on CSF EVPs were significantly decreased in the viral disease group (*p* = 0.005, *p* = 0.0151, and *p* = 0.0050, respectively; **Figures 5A-C**) compared to the non-viral disease group, although this trend is likely heavily influenced by the HAM patient CSF EVPs (open blue circles). The viral disease group demonstrated a specific increase in CD8+ and CD2+ CSF EVs (*p* = 0.0001 and *p* = 0.0027, respectively; **Figures 5D, E**) while showing no difference for CD4+ signals between groups (**Figure 5F**). Therefore, a combined dataset of EVPs in the CSF from all non-viral (n=26; HV, MS, and non-viral ONDs including: anti-NMDA receptor encephalitis, neurosarcoidosis, Susac syndrome, motor neuron disease, and lymphocytic variant hypereosinophilic syndrome) and viral infection [n= 23; HAM (open blue circles), AC, and viral ONDs including: PML, HSV-1 encephalitis, HSV-2 meningitis, Jamestown canyon virus, and HIV-1] samples was compiled (**Figure 6**). EVP tetraspanin signals were now not different between viral and non-viral groups, with median signals reaching 2.47 – 2.52 x 10^5^, 4.97 – 5.65 x 10^4^, and 5.01 – 5.40 x 10^4^ MESF units for CD81, CD63, and CD9, respectively (**Figures 6A-C**). Importantly, CD8+ and CD2+ CSF EVP signals continued to show significantly elevated signatures for the virally infected group in comparison to non-viral **(** *p* < 0.0001 for both, **Figures 6D, E**). CD4+ EVP signals remained similar between viral and non-viral groups (**Figure 6F**). When samples were grouped into viral vs. non-viral, EVP-associated CD40 and CD44 signals were elevated in those with virus infection (*p* = 0.0413 and *p* = 0.0176, respectively; **Figures 6G, H**). CD133/1 on EVPs from the virus infection group was decreased in comparison to non-viral (*p* = 0.0337, **Figure 6I**). All other markers measured and analyzed are shown (**Supplementary Figure 6**). Collectively, these data indicate that in a cohort of widely varied neurological diseases of viral and non-viral etiologies, consistent EVP-associated immune signatures were observed. CD8 and CD2 signatures on CSF EVPs from diseases linked with viruses were reproducibly shown to be elevated in comparison to non-viral diseases and controls, potentially indicating an EVP signature of viral-mediated disease in the CNS.

## Discussion

While many aspects of cellular immunological differences have been studied in the plasma and serum of individuals with HAM, MS, and other neurological diseases, no study to date has evaluated the EVP repertoires based on EVP surface receptor signatures within the CSF of these patients. Our results demonstrate CSF EVP signatures that reflect both the cellular origins of the EVPs and the pathophysiologic processes in the CNS of donors with viral neurological diseases.

We obtained CSF samples of well-characterized patient and control cohorts and utilized them for flow-based MPA of EVP surface markers. The MPA utilized a commercially available barcoded set of 39 different antibody-capture beads specific to various cell surface markers, including those from immunological, platelet, endothelial, and cancer-like cells, as well as from EV-enriched tetraspanins. MRPS analysis indicated that the concentrations of CSF EVPs from HV, HAM, and MS groups ranged from approximately 4 x 10^8^ to 1.5 x 10^9^ particles/mL (**Figure 2A**), with similar size and concentration distributions between clinical sample types. Although the tetraspanin markers were strongly positive across all samples, some minor differences were observed in the CSF EVP membrane-associated marker levels between sample groups. Specifically, HAM patient EVPs demonstrated decreased levels of CD81, CD63, and CD9, albeit not significantly from all disease groups (**Figures 2B-D**). This is consistent with reports that have demonstrated alterations in the tetraspanins of EVPs from virally infected cells, including those infected with HTLV-1 and closely-related retrovirus HIV-1 (27–29). Indeed, our previous study looking at EVPs from HAM patient PBMCs showed that there were differences in the profiles of CD63 compared to HV peripheral blood mononuclear cell EVPs (18). Therefore, it is possible that during pathogenesis of HAM, cells infected with HTLV-1 go through altered EVP biogenesis pathways, thereby resulting in EVPs with altered tetraspanin expression.

In this study, we identified a specific and significant increase in the signals of CSF EVPs expressing CD8 and CD2 in HAM, as compared to MS patients and HVs (**Figure 3**). Despite CD4+ T-cells remaining the preferred target for HTLV-1 infection, EVPs expressing CD4 were not significantly different between any clinical groups. Furthermore, the CD8+ EVPs from HAM patients were significantly positively correlated with both total cell number in the CSF and the number of CD8+ T-cells in the CSF (**Figures 4A, B**). These findings are consistent with the pathogenesis of HAM in which CD8+ T-cells are activated during infection, resulting in differentiation into HTLV-1-specific CD8+ CTLs, which then migrate into the CNS (7, 8, 26). It is important to consider that the concentrations of CD8+ T-cells in the CSF of our cohort ranged widely from undetectable to 1.12 x 10^4^ cells/mL (data not shown), whereas we were able to detect levels of CD8+ EVP signals between 1 x 10^3^ to 5 x 10^4^ MESF units using 250 µL of CSF (corresponding to on average ~2.25 x 10^8^ EVPs based on MRPS measurements; **Figure 2A**). Therefore, detecting T-cell signals on EVPs in the CSF by MPA was maybe more sensitive than standard flow cytometry analyses that must rely on cells.

CD8+ CTLs in the CNS produce and release several pro-inflammatory cytokines including interferon-γ (IFNγ) and tumor necrosis factor-α (TNF-α), which is proposed to result in substantial bystander destruction of resident CNS tissue and neural cells associated with demyelination (7, 30). Moreover, several past studies have shown that EVs from HTLV-1-infected cells contain HTLV-1 Tax protein and other viral components and are capable of functionally impacting the spread of infection and inflammation to recipient cells of various types (18, 31–34). As such, the significance of being able to detect increases in CD8+ and CD2+ EVPs with HAM compared to HVs and MS patients in the CSF may go beyond a potential novel biomarker – it is possible that the EVPs themselves may play a role in HAM pathogenesis. It is known that EVPs from T-cells can function in many ways, including as 1) antigen presenters and co-stimulators to aid activation of immune cells, 2) inhibitors of T-cell activation, and, in the case of EVPs from activated CD8+ cells, as 3) cytotoxic vehicles (35–37). It will be of great interest to explore the functional effects and cargoes of the upregulated EVP sub-populations in HAM patient CSF compared to other viral and non-viral neurological diseases. In addition to HAM, elevated levels of CD8+ and CD2+ CSF EVPs were demonstrated in a limited number of virally associated chronic neurological disease carriers compared to patients with non-viral mediated neurologic disease (**Figures 5**, **6D, E**). This was in contrast to the levels of CD4+ EVPs, which did not show any significant differences between viral and non-viral diseases (**Figures 5**, **6F**). Certainly, a larger group of patients with viral-associated neurologic disease will be needed to determine if the observation of CSF EVPs derived from CD8+ T-cells maybe a useful biomarker of CNS viral infection.

We found that EVP CD44 and CD40 markers were also increased in CSF samples from individuals with viral neurological diseases in our cohort and in CSF samples from asymptomatic carriers of HTLV-1 (**Figures 6G, H**). CD40 is a well-known marker of B-cells but is also expressed on monocytes and antigen presenting cells (APCs). CD40+ CSF EVPs may be increased in viral diseases since CD40+ cells are commonly activated and increased in the CNS during infection. Indeed, CD40-expressing EVs in the plasma have previously been found to be significantly correlated with HIV viral load (38). CD44 is a ubiquitous cell surface adhesion receptor that has been shown to be a marker of some stem cells, metastatic cancer cells, and interestingly, memory T-cells (39, 40). In its membrane-bound form, it binds extracellular matrix ligands such as hyaluronic acid (HA) which can result in many outcomes, including anti-inflammatory effects, inhibition of activation/proliferation, or can stimulate inflammatory responses in macrophages or death of activated T-cells (41). The observation of increased CD40+ and CD44+ EVPs in the CSF may be an important and useful marker to differentiate between chronic neurological diseases caused by viruses and those of non-viral origin. Furthermore, EVPs in the CSF with CD40 or CD44 present on their surface may potentially play functional roles in the immunopathogenesis of neurological diseases of viral etiology. For example, CD44+ CSF EVPs elevated in viral infection may have possible activity as an adhesion molecule to aid in the migration of lymphocytes to the CNS (42). Additionally, CD40+ CSF EVPs, potentially originating from B-cells or APCs, may play critical roles in antigen presentation and the development of a mature immune response against CNS-invading viruses.

Finally, we found that CD133/1+ EVPs in the CSF were significantly decreased in HAM patients compared to MS patients (**Figure 3D**). This trend was also observed in CSF EVPs from patients with other viral diseases, which also had significantly less EVP-associated CD133/1 (**Figure 6I**). CD133/1 (prominin-1) is highly expressed in hematopoietic progenitor cells, cancer stem cells, neural stem cells, and others (24, 25). Increased EVPs with CD133 have previously been observed in the CSF of MS and especially glioblastoma patients, but their function remains poorly understood (43, 44). It was hypothesized that their release is linked to the differentiation of stem and cancer cells (44). We do not yet know from which cell type(s) the CD133/1+ CSF EVPs originated in this study, however, CSF EVPs with CD133/1 were inversely correlated with CD8+ EVs (**Figure 4C**). We hypothesize that during CNS viral infection there may be an increase in hematopoietic and neural stem cell differentiation, resulting in a decrease in CD133/1+ cells and a corresponding increase in the number of more mature, potentially inflammatory cell types (i.e. CD8+ CTLs) to respond to the infection. Future study is required to address this hypothesis.

Here, we have optimized and performed MPAs of various neurological disease group CSF samples to determine EVP surface marker differences between pathologies. We have demonstrated higher levels of CD8 and CD2 on CSF EVPs from patients with virus-associated neurologic diseases and infections in this cohort. There are some potential limitations we acknowledge with this work. It is possible that containing our analysis to 39 surface markers that we are excluding several important and biologically relevant other signatures on CSF EVPs. Neural-specific markers such as L1CAM, MOG, MBP, GLAST, and neurofilament, which were not commercially available in a comparable kit at the time of this study, will be of particular interest to investigate going forward. Several studies have shown compelling evidence that viruses, particularly Epstein-Barr virus (EBV), may act as a trigger for MS through interaction with genetic and other factors (45, 46); however, it is unlikely that a single virus is the etiologic mediator of this disorder (47–49). If indeed CD8+ EVPs in CSF are a marker of viral infection, the results in this study did not demonstrate CSF EVPs from MS with a viral immune profile as was observed in other virus-associated chronic neurologic diseases. In this study we did not stratify immune-mediated diseases based on degree of active inflammation due to limited sample sizes. Future studies may need to address the effects of active inflammation on CSF EVPs. Regardless, it is well known that subsets of CD8+ T cells are cytotoxic and function to recognize and eliminate cells infected with virus (35–37). Substantially elevated levels of CD8+ T-cell markers on CSF EVPs from patients with HAM disease or other viral infections are consistent with the hypothesis that these CSF EVPs are derived from virus-specific adaptive immune responses and thus may represent a signature of viral infection in the CNS. Additionally, EVPs originating from T-cells have been shown in many circumstances to impact the activation of inflammatory responses or induction of apoptosis in recipient immune cells.

Since many neurological diseases caused by viruses, including HAM, involve the infiltration of CD8+ CTLs into the CNS, it is possible that EVPs originating from activated CD8+ T-cells may play important functional roles in the neural proinflammatory response, both through antigen presentation and activation of other immune cell types, and/or by inducing inflammation, cytokine production, and cell death in neural lineage cells. These data collectively suggest that CD8+ and CD2+ EVPs may be significant in two respects: 1) CSF EVPs possessing CD8 and/or CD2 may be used as a potential biomarker for viral-mediated neurological diseases such as HAM, and 2) CD8+ and/or CD2+ EVPs may themselves mediate areas of the disease process in infected individuals. Ultimately, as methods for evaluating these EVP subsets and their cargo improve in sensitivity and analytical depth, these CD8 and CD2 EVP populations may be useful for monitoring treatment responses or disease activity in patients with chronic viral CNS conditions.

## Data availability statement

The original contributions presented in the study are included in the article/**Supplementary Material**. Further inquiries can be directed to the corresponding authors.

## Ethics statement

The studies involving humans were approved by National Institutes of Health Institutional Review Board. The studies were conducted in accordance with the local legislation and institutional requirements. The participants provided their written informed consent to participate in this study.

## Author contributions

MP and JW performed the experiments, data analysis, and wrote the manuscript. ES, SC, D-AJ, BK, and TT helped with portions of experiments and optimization of experimental pipelines. AC, RH, MM, NN, JO, YE-A, AN, IC, and DR were responsible for patient sample acquisition, storage, and research-based and clinical data procurement from patients at the NIH clinical center. JJ and SJ oversaw all experimental designs, sample usage, and overall direction of the manuscript. All authors contributed to the article and approved the submitted version.

## References

[1] Futsch N Mahieux R Dutartre H. HTLV-1, the other pathogenic yet neglected human retrovirus: from transmission to therapeutic treatment. Viruses (2017) 10:1. doi: 10.3390/v10010001 29267225 PMC5795414

[2] Howard AK Li DKB Oger J. MRI contributes to the differentiation between MS and HTLV-I associated myelopathy in british columbian coastal natives. Can J Neurol Sci/J Canadien Des Sci Neurol (2003) 30:41–8. doi: 10.1017/S0317167100002420 12619783

[3] Proietti FA Carneiro-Proietti ABF Catalan-Soares BC Murphy EL. Global epidemiology of HTLV-I infection and associated diseases’. Oncogene (2005) 24:6058–68. doi: 10.1038/sj.onc.1208968 16155612

[4] Puccioni-Sohler M Yamano Y Rios M Carvalho SMF Vasconcelos CCF Papais-Alvarenga R. Differentiation of HAM/TSP from patients with multiple sclerosis infected with HTLV-I’. Neurology (2007) 68:206–13. doi: 10.1212/01.wnl.0000251300.24540.c4 17224575

[5] Yamano Y Cohen CJ Takenouchi N Yao K Tomaru U Li H-C. Increased expression of human T lymphocyte virus type I (HTLV-I) tax11-19 peptide–human histocompatibility leukocyte antigen A*201 complexes on CD4+ CD25+T cells detected by peptide-specific, major histocompatibility complex–restricted antibodies in patie’. J Exp Med (2004) 199:1367–77. doi: 10.1084/jem.20032042 PMC2211812 15136590

[6] Bangham CRM. ‘CTL quality and the control of human retroviral infections. Eur J Immunol (2009) 39:1700–12. doi: 10.1002/eji.200939451 19582737

[7] Jacobson S Shida H Mcfarlin DE Fauci AS Koenig S. Circulating CD8+ cytotoxic T lymphocytes specific for HTLV-I pX in patients with HTLV-I associated neurological disease. Nature (1990) 348:245–48. doi: 10.1038/348245a0 2146511

[8] Kubota R Furukawa Y Izumo S Usuku K Osame M. Degenerate specificity of HTLV-1–specific CD8+ T cells during viral replication in patients with HTLV-1–associated myelopathy (HAM/TSP). Blood (2003) 101:3074–81. doi: 10.1182/blood-2002-08-2477 12480698

[9] Rende F Cavallari I Corradin A Silic-Benussi M Toulza F Toffolo GM. ‘Kinetics and intracellular compartmentalization of HTLV-1 gene expression: nuclear retention of HBZ mRNAs’. Blood (2011) 117:4855–59. doi: 10.1182/blood-2010-11-316463 PMC5292588 21398577

[10] Wouk J Rechenchoski DZ Rodrigues BCD Ribelato EV Faccin-Galhardi LC. Viral infections and their relationship to neurological disorders’. Arch Virol (2021) 166:733–53. doi: 10.1007/s00705-021-04959-6 PMC7838016 33502593

[11] Baecher-Allan C Kaskow BJ Weiner HL. ‘Multiple sclerosis: mechanisms and immunotherapy. Neuron (2018) 97:742–68. doi: 10.1016/j.neuron.2018.01.021 29470968

[12] Fierz W. ‘Multiple sclerosis: an example of pathogenic viral interaction? Virol J (2017) 14:42. doi: 10.1186/s12985-017-0719-3 28241767 PMC5330019

[13] Shah R Patel T Freedman JE. Circulating extracellular vesicles in human disease’. New Engl J Med (2018) 379:958–66. doi: 10.1056/NEJMra1704286 30184457

[14] Wu WC Song SJ Zhang Y Li X. Role of extracellular vesicles in autoimmune pathogenesis’. Front Immunol (2020) 11. doi: 10.3389/fimmu.2020.579043 PMC7538611 33072123

[15] Alenquer M Amorim M. Exosome biogenesis, regulation, and function in viral infection. Viruses (2015) 7:5066–83. doi: 10.3390/v7092862 PMC4584306 26393640

[16] Anderson MR Kashanchi F Jacobson S. Exosomes in viral disease. Neurotherapeutics (2016) 13:535–46. doi: 10.1007/s13311-016-0450-6 PMC4965413 27324390

[17] Pleet ML Branscome H DeMarino C Pinto DO Zadeh MA Rodriguez M. Autophagy, EVs, and infections: A perfect question for a perfect time’. Front Cell Infect Microbiol (2018) 8:362. doi: 10.3389/fcimb.2018.00362 30406039 PMC6201680

[18] Anderson MR Pleet ML Enose-Akahata Y Erickson J Monaco MC Akpamagbo Y. Viral antigens detectable in CSF exosomes from patients with retrovirus associated neurologic disease: functional role of exosomes. Clin Trans Med (2018) 7(1):24. doi: 10.1186/s40169-018-0204-7 PMC6110307 30146667

[19] Enose-Akahata Y Azodi S Smith BR Billioux BJ Vellucci A Ngouth N. Immunophenotypic characterization of CSF B cells in virus-associated neuroinflammatory diseases. PloS Pathog (2018) 14:e1007042. doi: 10.1371/journal.ppat.1007042 29709026 PMC5945224

[20] Welsh JA Jones JC Tang VA. Fluorescence and light scatter calibration allow comparisons of small particle data in standard units across different flow cytometry platforms and detector settings’. Cytometry Part A (2020) 97:592–601. doi: 10.1002/cyto.a.24029 PMC8482305 32476280

[21] Welsh JA Horak P Wilkinson JS Ford VJ Jones JC Smith D. FCMPASS software aids extracellular vesicle light scatter standardization’. Cytometry Part A (2020) 97:569–81. doi: 10.1002/cyto.a.23782 PMC7061335 31250561

[22] Welsh JA Jones JC. Small particle fluorescence and light scatter calibration using FCMPASS software. Curr Protoc Cytometry (2020) 94(1):e79. doi: 10.1002/cpcy.79 PMC8623744 32936529

[23] Welsh JA Killingsworth B Kepley J Traynor T Cook S Savage J. MPAPASS software enables stitched multiplex, multidimensional EV repertoire analysis and a standard framework for reporting bead-based assays’. Cell Rep Methods (2022) 2:100136. doi: 10.1016/j.crmeth.2021.100136 35474866 PMC9017130

[24] Aghajani M Mansoori B Mohammadi A Asadzadeh Z Baradaran B. New emerging roles of CD133 in cancer stem cell: Signaling pathway and miRNA regulation. J Cell Physiol (2019) 234:21642–61. doi: 10.1002/jcp.28824 31102292

[25] Wang H Gong P Li J Fu Y Zhou Z Liu L. Role of CD133 in human embryonic stem cell proliferation and teratoma formation. Stem Cell Res Ther (2020) 183(6):857–64. doi: 10.1186/s13287-020-01729-0 PMC7251672 32460847

[26] Ureta-Vidal A Pique C Garcia Z Dehée A Tortevoye P Désiré N. Human T cell leukemia virus type I (HTLV-I) infection induces greater expansions of CD8 T lymphocytes in persons with HTLV-I–associated myelopathy/tropical spastic paraparesis than in asymptomatic carriers’. J Infect Dis (2001) 183:857–64. doi: 10.1086/319264 11237801

[27] Narayanan A Iordanskiy S Das R Duyne RV Santos S Jaworski E. Exosomes derived from HIV-1-infected cells contain trans-activation response element RNA. J Biol Chem (2013) 288:20014–33. doi: 10.1074/jbc.M112.438895 PMC3707700 23661700

[28] Madison M Okeoma C. Exosomes: implications in HIV-1 pathogenesis. Viruses (2015) 7:4093–118. doi: 10.3390/v7072810 PMC4517139 26205405

[29] Sampey GC Saifuddin M Schwab A Barclay R Punya S Chung M-C. Exosomes from HIV-1-infected cells stimulate production of pro-inflammatory cytokines through trans-activating response (TAR) RNA’. J Biol Chem (2016) 291:1251–66. doi: 10.1074/jbc.M115.662171 PMC4714213 26553869

[30] Nozuma S Kubota R Jacobson S. Human T-lymphotropic virus type 1 (HTLV-1) and cellular immune response in HTLV-1-associated myelopathy/tropical spastic paraparesis. J Neurovirol (2020) 26:652–63. doi: 10.1007/s13365-020-00881-w PMC7532128 32705480

[31] Jaworski E Narayanan A Duyne RV Shabbeer-Meyering S Iordanskiy S. Human T-lymphotropic virus type 1-infected cells secrete exosomes that contain tax protein. J Biol Chem (2014) 289:22284–305. doi: 10.1074/jbc.M114.549659 PMC4139239 24939845

[32] Barclay RA Pleet ML Akpamagbo Y Noor K Mathiesen A Kashanchi F. Isolation of exosomes from HTLV-infected cells. Methods Mol Biol (2017) 1582:57–75. doi: 10.1007/978-1-4939-6872-5_5 28357662

[33] Pinto DO DeMarino C Pleet ML Cowen M Branscome H Al Sharif S. ‘HTLV-1 extracellular vesicles promote cell-to-cell contact. Front Microbiol (2019) 10:2147. doi: 10.3389/fmicb.2019.02147 31620104 PMC6759572

[34] Al Sharif S Pinto DO Mensah GA Dehbandi F Khatkar P Kim Y. Extracellular vesicles in HTLV-1 communication: the story of an invisible messenger. Viruses (2020) 12:1422. doi: 10.3390/v12121422 33322043 PMC7763366

[35] Gutiérrez-Vázquez C Villarroya-Beltri C Mittelbrunn M Sánchez-Madrid F. Transfer of extracellular vesicles during immune cell-cell interactions. Immunol Rev (2013) 251:125–42. doi: 10.1111/imr.12013 PMC3740495 23278745

[36] Lindenbergh MFS Stoorvogel W. Antigen presentation by extracellular vesicles from professional antigen-presenting cells. Annu Rev Immunol (2018) 36:435–59. doi: 10.1146/annurev-immunol-041015-055700 29400984

[37] Seo N Shirakura Y Tahara Y Momose F Harada N Ikeda H. Activated CD8+ T cell extracellular vesicles prevent tumour progression by targeting of lesional mesenchymal cells. Nat Commun (2018) 9(1):435. doi: 10.1038/s41467-018-02865-1 PMC5789986 29382847

[38] Martínez LE Lensing S Chang D Magpantay LI Mitsuyasu R Ambinder RF. Plasma extracellular vesicles bearing PD-L1, CD40, CD40L or TNF-RII are significantly reduced after treatment of AIDS-NHL. Sci Rep (2022) 12(1):9185. doi: 10.1038/s41598-022-13101-8 PMC9163074 35655072

[39] Baaten BJ Tinoco R Chen AT Bradley LM. Regulation of antigen-experienced T cells: lessons from the quintessential memory marker CD44. Front Immunol (2012) 3:23. doi: 10.3389/fimmu.2012.00023 22566907 PMC3342067

[40] Senbanjo LT Chellaiah MA. ‘CD44: A multifunctional cell surface adhesion receptor is a regulator of progression and metastasis of cancer cells’. Front Cell Dev Biol (2017) 5:18. doi: 10.3389/fcell.2017.00018 28326306 PMC5339222

[41] Szatanek R Baj-Krzyworzeka M. CD44 and tumor-derived extracellular vesicles (TEVs). Possible gateway to cancer metastasis’. Int J Mol Sci (2021) 22:1463. doi: 10.3390/ijms22031463 33540535 PMC7867195

[42] Jordan AR Racine RR Hennig MJ Lokeshwar VB. ‘The role of CD44 in disease pathophysiology and targeted treatment. Front Immunol (2015) 6:182. doi: 10.3389/fimmu.2015.00182 25954275 PMC4404944

[43] Huttner HB Janich P Kohrmann M Jaszai J Siebzehnrubl F Blumcke I. ‘The stem cell marker prominin-1/CD133 on membrane particles in human cerebrospinal fluid offers novel approaches for studying central nervous system disease. Stem Cells (2008) 26:698–705. doi: 10.1634/stemcells.2007-0639 18096722

[44] Marzesco A-M. Prominin-1-containing membrane vesicles: origins, formation, and utility. In: Advances in Experimental Medicine and Biology. New York: Springer (2013). 10.1007/978-1-4614-5894-4_3 23161074

[45] Jacobs BM Giovannoni G Cuzick J Dobson R. ‘Systematic review and meta-analysis of the association between Epstein–Barr virus, multiple sclerosis and other risk factors. Multiple Sclerosis J (2020) 26:1281–97. doi: 10.1177/1352458520907901 PMC7543008 32202208

[46] Bjornevik K Cortese M Healy BC Kuhle J Mina MJ Leng Y. Longitudinal analysis reveals high prevalence of Epstein-Barr virus associated with multiple sclerosis. Science (2022) 375:296–301. doi: 10.1126/science.abj8222 35025605

[47] Tarlinton R Khaibullin T Granatov E Martynova E Rizvanov A Khaiboullina S. The interaction between viral and environmental risk factors in the pathogenesis of multiple sclerosis’. Int J Mol Sci (2019) 20:303. doi: 10.3390/ijms20020303 30646507 PMC6359439

[48] Tarlinton RE Martynova E Rizvanov AA Khaiboullina S Verma S. Role of viruses in the pathogenesis of multiple sclerosis’. Viruses (2020) 12:643. doi: 10.3390/v12060643 32545816 PMC7354629

[49] Sedighi S Gholizadeh O Yasamineh S Akbarzadeh S Amini P Favakehi P. Comprehensive investigations relationship between viral infections and multiple sclerosis pathogenesis. Curr Microbiol (2023) 80(1):15. doi: 10.1007/s00284-022-03112-z PMC9716500 36459252

