# Supplementary material for: Viral Immune signatures from cerebrospinal fluid extracellular vesicles and particles in HAM and other chronic neurological diseases
Source: Front Immunol. 2023 Aug 9;14:1235791. doi: 10.3389/fimmu.2023.1235791 (PMC10446883; doi:10.3389/fimmu.2023.1235791)
Supplement: Supplementary file 1 [file DataSheet_1.pdf]

Supplementary Material

Viral Immune Signatures from Cerebrospinal Fluid Extracellular Vesicles and Particles in HAM and Other Chronic Neurological Diseases

Michelle L. Pleet[1†], Joshua A. Welsh[2†], Emily H. Stack[1], Sean Cook[2], Dove-Anna Johnson[2], Bryce Killingsworth[2], Tim Traynor[2], Annaliese Clauze[1], Randall Hughes[1], Maria Chiara Monaco[1], Nyater Ngouth[1], Joan Ohayon[1], Yoshimi Enose-Akahata[1], Avindra Nath[3], Irene Cortese[4], Daniel S. Reich[5], Jennifer C. Jones[2T*], Steven Jacobson[1T*]

[1]Viral Immunology Section, Neuroimmunology Branch, National Institute of Neurological Disorders and Stroke, National Institutes of Health, Bethesda, MD, USA

[2] Translational Nanobiology Section, Laboratory of Pathology, Center for Cancer Research, National Cancer Institute, National Institutes of Health, Bethesda, MD, USA

[3] Section of Infections of the Nervous System, National Institute of Neurological Disorders and Stroke, National Institutes of Health, Bethesda, MD, USA

[4] Experimental Immunotherapeutics Unit, National Institute of Neurological Disorders and Stroke, National Institutes of Health, Bethesda, MD, USA

[5] Translational Neuroradiology Section, National Institute of Neurological Disorders and Stroke, National Institutes of Health, Bethesda, MD, USA

[†] both authors contributed equally

[T] both senior authors contributed equally

*** Correspondence:**
Steven Jacobson, Ph.D. and Jennifer C. Jones, M.D., Ph.D.
jacobsons@nih.gov and jennifer.jones2@nih.gov

Supplementary Figures and Table

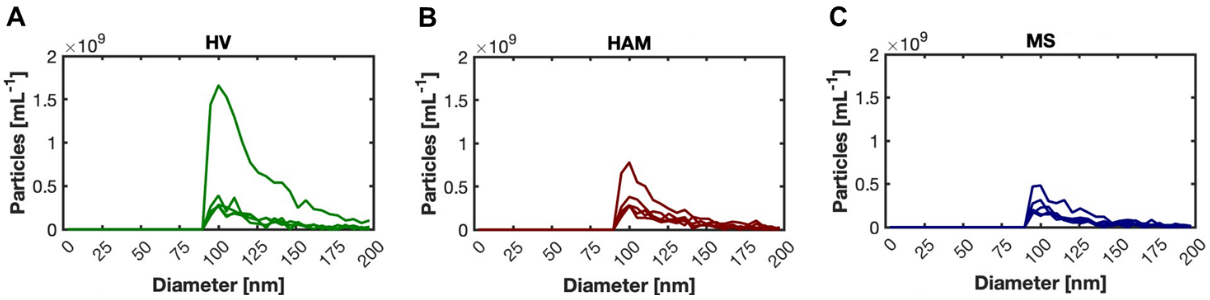

Supplementary Figure 1. Representative sizing data from healthy volunteer (HV), HTLV-1-associated myelopathy (HAM), and multiple sclerosis (MS) CSF samples (n=5 each) measured by MRPS (Spectradyne, nCS1). Raw data was normalized using NIST spike-in beads in MATLAB. Normalized size distribution outputs are shown overlaid for each clinical group: **A)** HV, **B)** HAM, and **C)** MS.

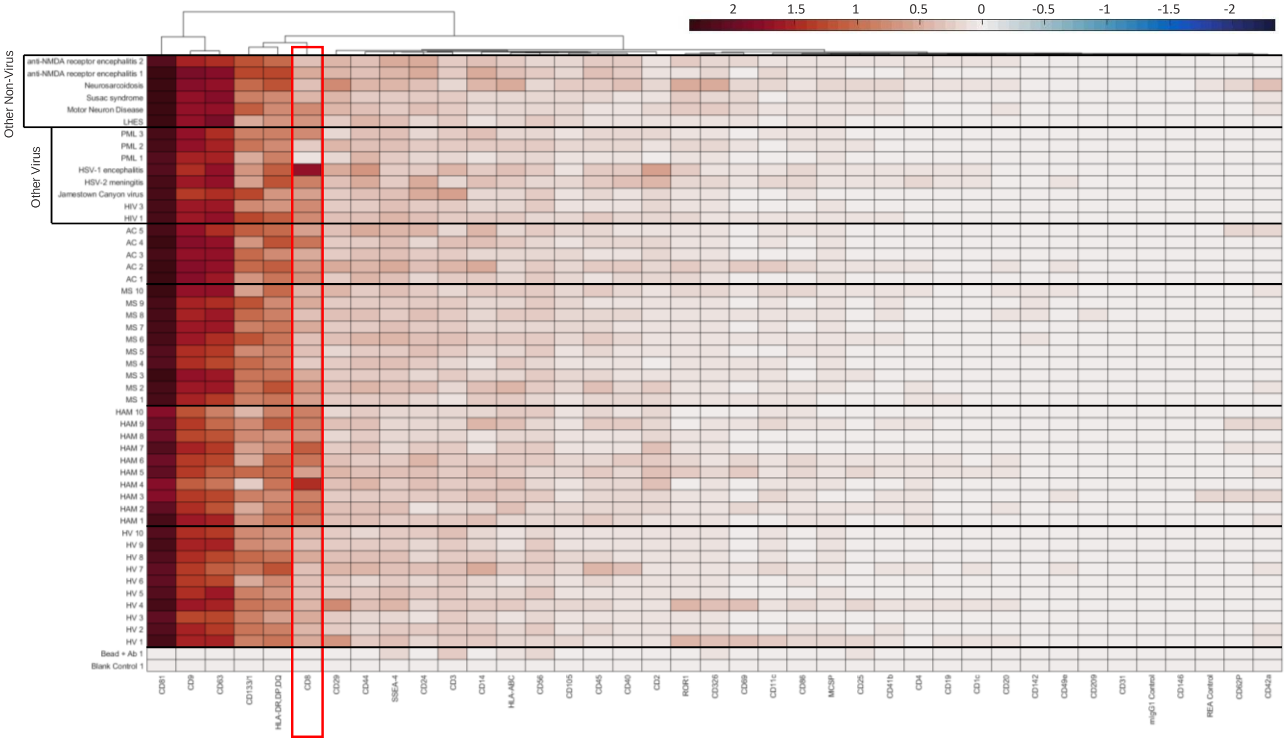

Supplemental Figure 2: Heatmap of normalized MPA data output. Highest titrations (250 µL) of each sample are shown, grouped by disease category (left axis) along with representative blank control (capture beads alone) and bead + Ab control (capture beads incubated with detection antibodies and no EVP sample; bottom two rows). Sample data normalized to the blank control using MPA$_{PASS}$ software are shown as a fold change on log 10 scale. CD8+ EVP signals are boxed in red.

A

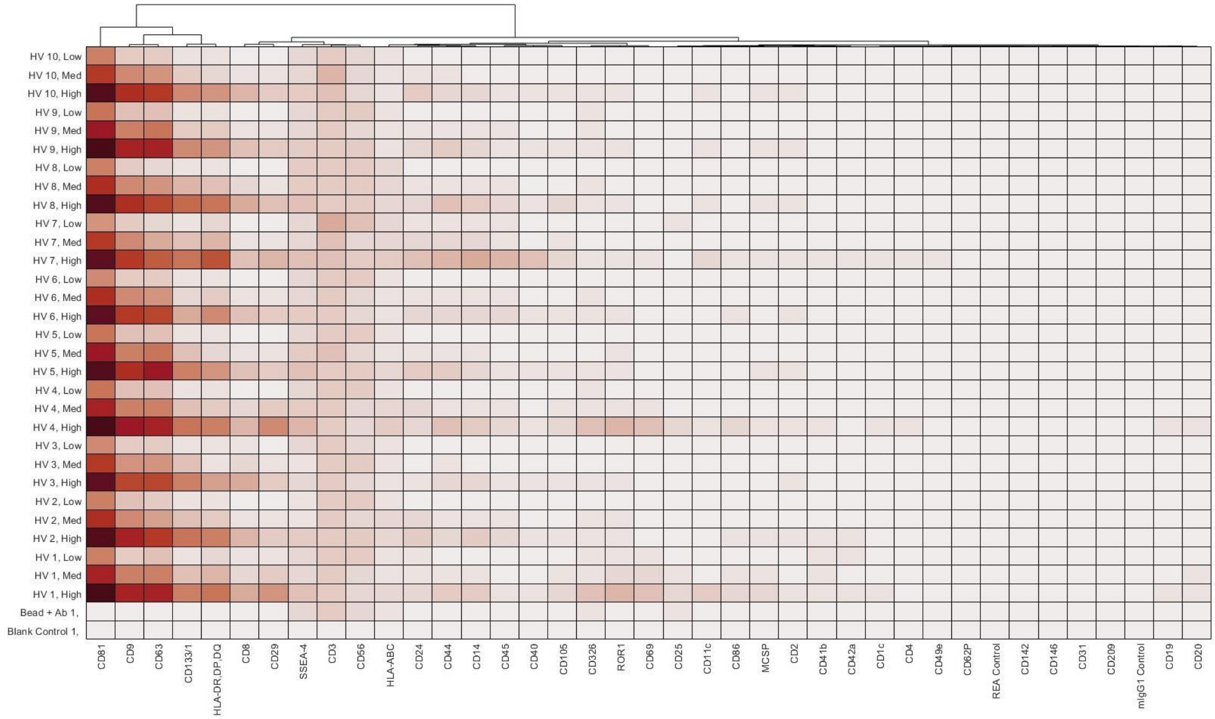
HV 10, Low
HV 10, Med
HV 10, High
HV 9, Low
HV 9, Med
HV 9, High
HV 8, Low
HV 8, Med
HV 8, High
HV 7, Low
HV 7, Med
HV 7, High
HV 6, Low
HV 6, Med
HV 6, High
HV 5, Low
HV 5, Med
HV 5, High
HV 4, Low
HV 4, Med
HV 4, High
HV 3, Low
HV 3, Med
HV 3, High
HV 2, Low
HV 2, Med
HV 2, High
HV 1, Low
HV 1, Med
HV 1, High
Bead + Ab 1,
Blank Control 1,
CD81
CD9
CD63
CD133/1
HLA-DR,DP,DQ
CD8
CD29
SSEA-4
CD3
CD56
HLA-ABC
CD24
CD44
CD14
CD45
CD40
CD105
CD326
ROR1
CD69
CD25
CD11c
CD86
MCSP
CD2
CD41b
CD42a
CD1c
CD4
CD49e
CD62P
REA Control
CD142
CD146
CD31
CD209
mIgG1 Control
CD19
CD20

B

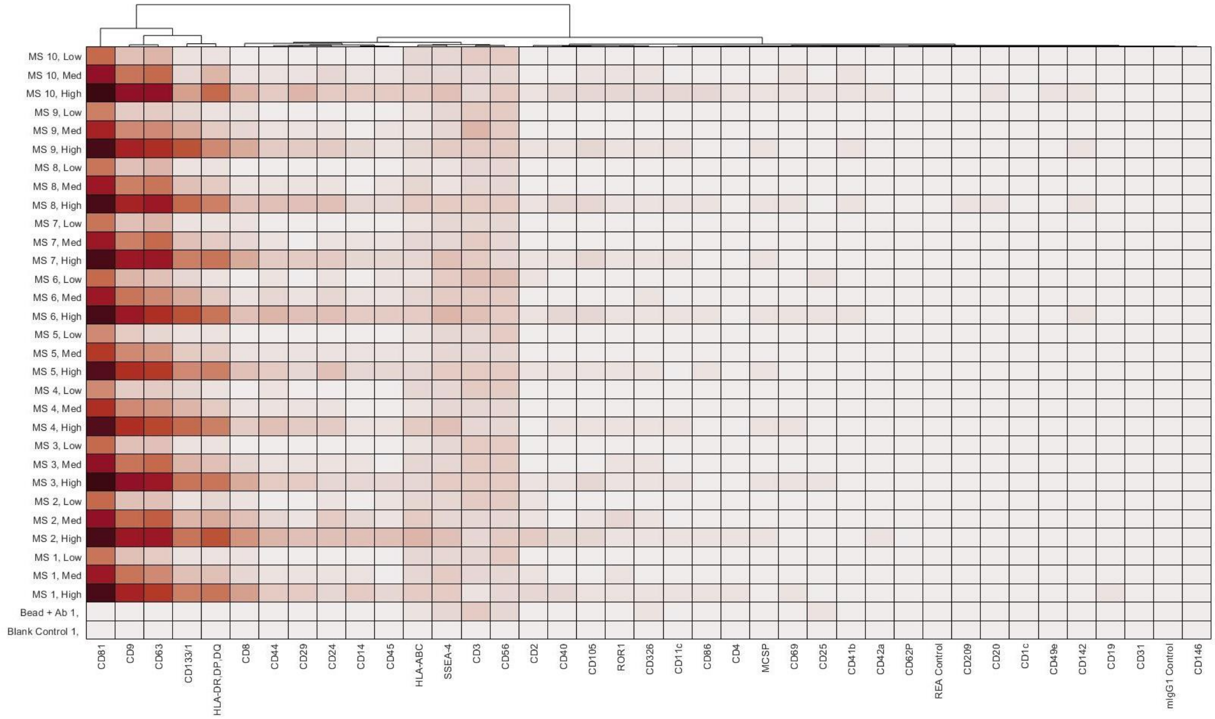
MS 10, Low
MS 10, Med
MS 10, High
MS 9, Low
MS 9, Med
MS 9, High
MS 8, Low
MS 8, Med
MS 8, High
MS 7, Low
MS 7, Med
MS 7, High
MS 6, Low
MS 6, Med
MS 6, High
MS 5, Low
MS 5, Med
MS 5, High
MS 4, Low
MS 4, Med
MS 4, High
MS 3, Low
MS 3, Med
MS 3, High
MS 2, Low
MS 2, Med
MS 2, High
MS 1, Low
MS 1, Med
MS 1, High
Bead + Ab 1,
Blank Control 1,
CD81
CD9
CD63
CD133/1
HLA-DR,DP,DQ
CD8
CD44
CD29
CD24
CD14
CD45
HLA-ABC
SSEA-4
CD3
CD56
CD2
CD40
CD105
ROR1
CD326
CD11c
CD86
CD4
MCSP
CD69
CD25
CD41b
CD42a
CD62P
REA Control
CD209
CD20
CD1c
CD49e
CD142
CD19
CD31
mIgG1 Control
CD146

C

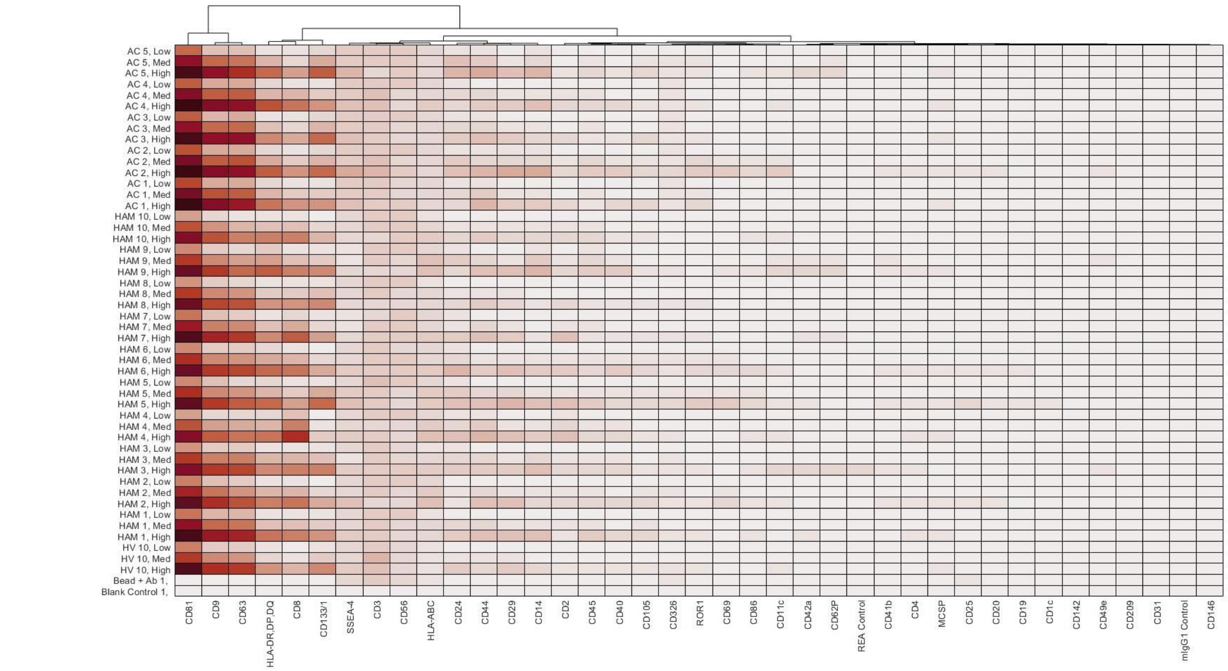

D

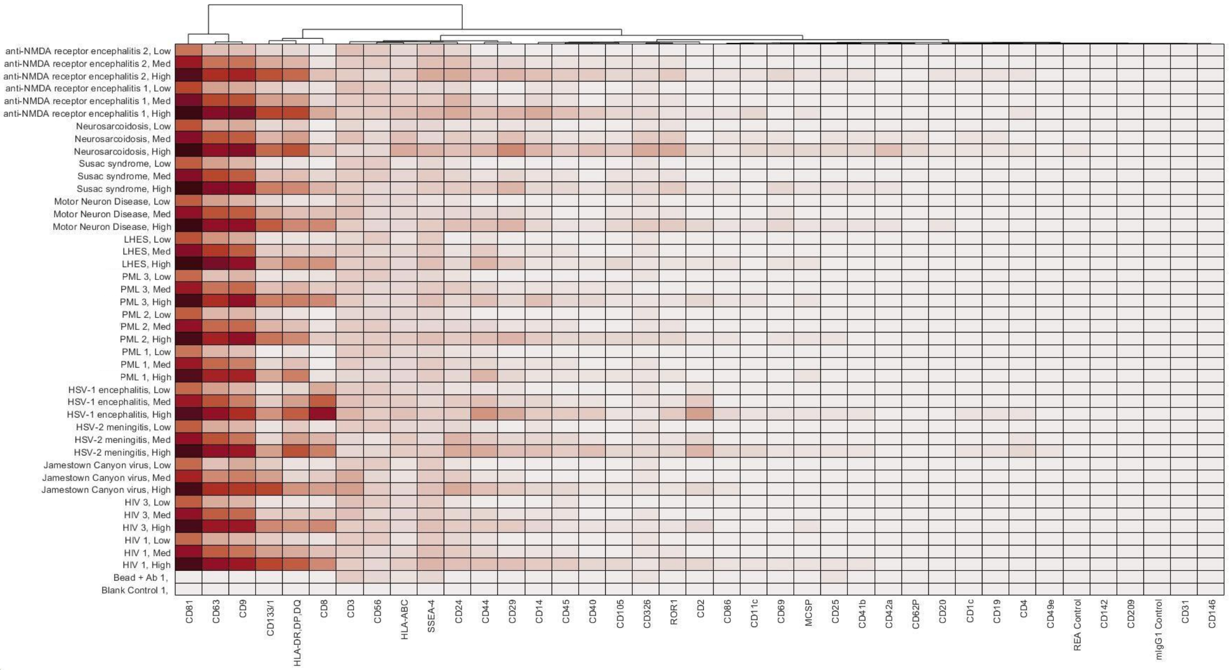

Supplemental Figure 3: Heatmap of normalized MPA data outputs in titration. All titrations (High = 250 µL; Med = 150 µL; Low = 50 µL) of HV **(A)**, MS **(B)**, HAM and AC **(C)**, and OND **(D)** samples are shown, grouped by disease category (left axis) along with representative blank control (capture beads alone) and bead + Ab control (capture beads incubated with detection antibodies and no EVP sample; bottom two rows). Samples were normalized to the blank control and are shown as a fold change on log 10 scale.

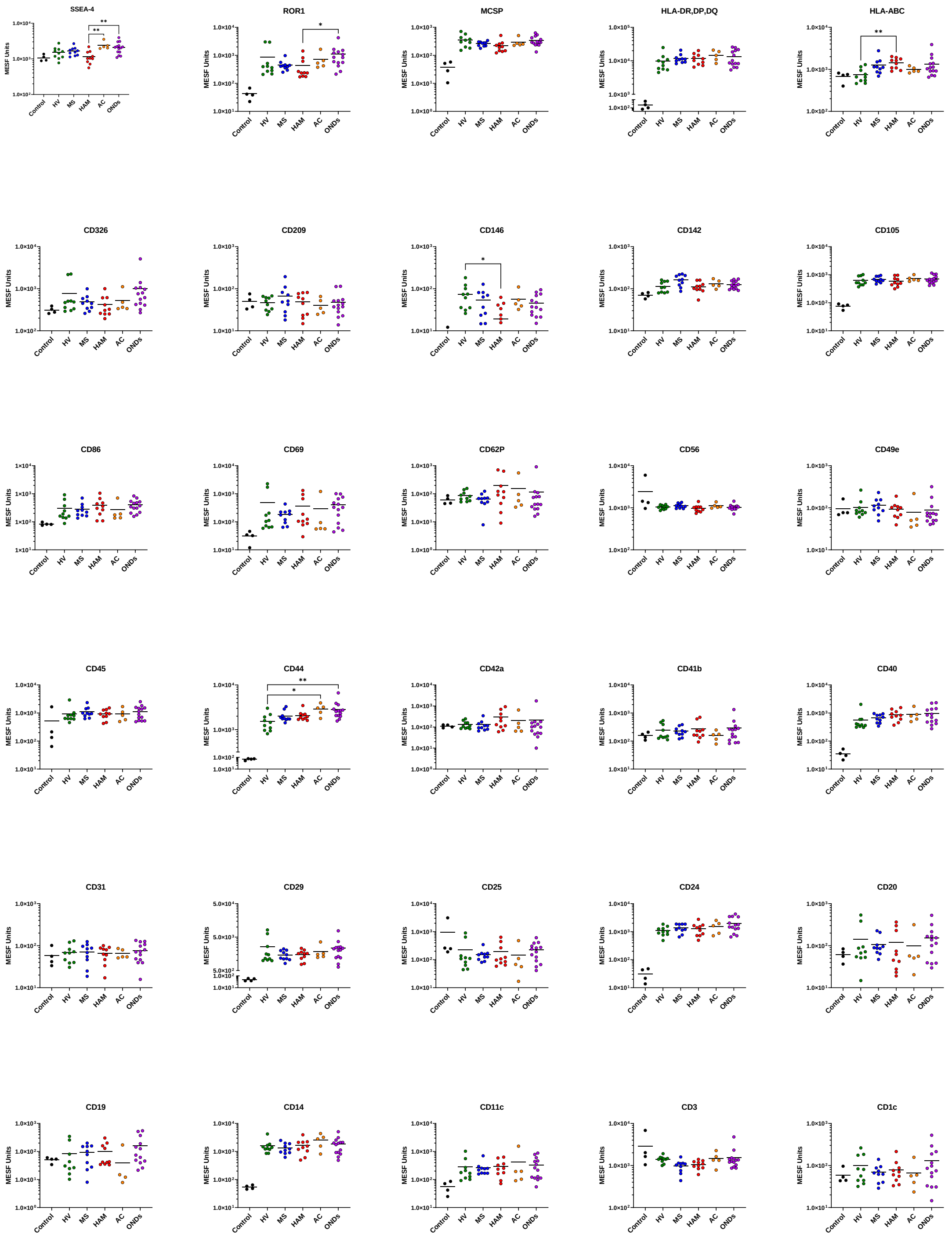

Supplemental Figure 4: MPA of additional CSF EVP surface markers compared by disease group. All additional MPA results of CSF EVPs from HV, MS, HAM (HAM), AC, and ONDs are shown. Results were normalized and converted from .fcs file arbitrary unit scales to APC MESF units using reference standards and MPA$_{PASS}$ software. Controls shown are the same capture beads incubated with detection antibodies in the absence of EVP sample, representing the nonspecific background fluorescence of the assay. Nonparametric statistical analyses comparing groups were performed using Kruskal-Wallis with Dunn's multiple comparisons tests ($p \leq 0.05$ = *; $p < 0.01$ = **).

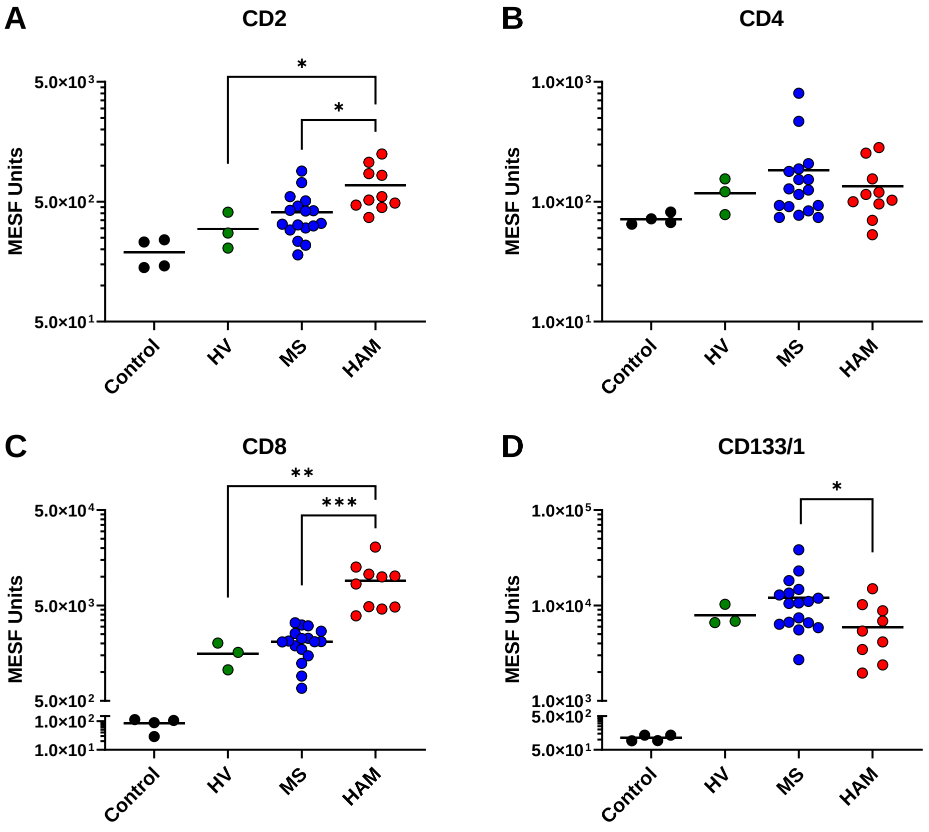

Supplemental Figure 5: Validation MPA of additional HV, HAM, and MS CSF samples. Additional MPA results of CSF EVPs with positive signals for CD2 (**A**), CD4 (**B**), CD8 (**C**), and CD133/1 (**D**) from HV (n=3), MS (n=17), HAM (HAM; n=10) are shown (**Supplemental Table 1**). A new MACSPlex Exosome Kit with a different lot number was used for this assay, in order to determine assay reproducibility and disease group specificity. Results were normalized and converted from .fcs file arbitrary unit scales to APC MESF units using reference standards and MPA_{PASS} software. Controls shown are the same capture beads incubated with detection antibodies in the absence of EVP sample, representing the nonspecific background fluorescence of the assay. Nonparametric statistical analyses comparing groups were performed using Kruskal-Wallis with Dunn's multiple comparisons tests ($p \leq 0.05$ = *; $p < 0.01$ = **; $p < 0.001$ = ***).

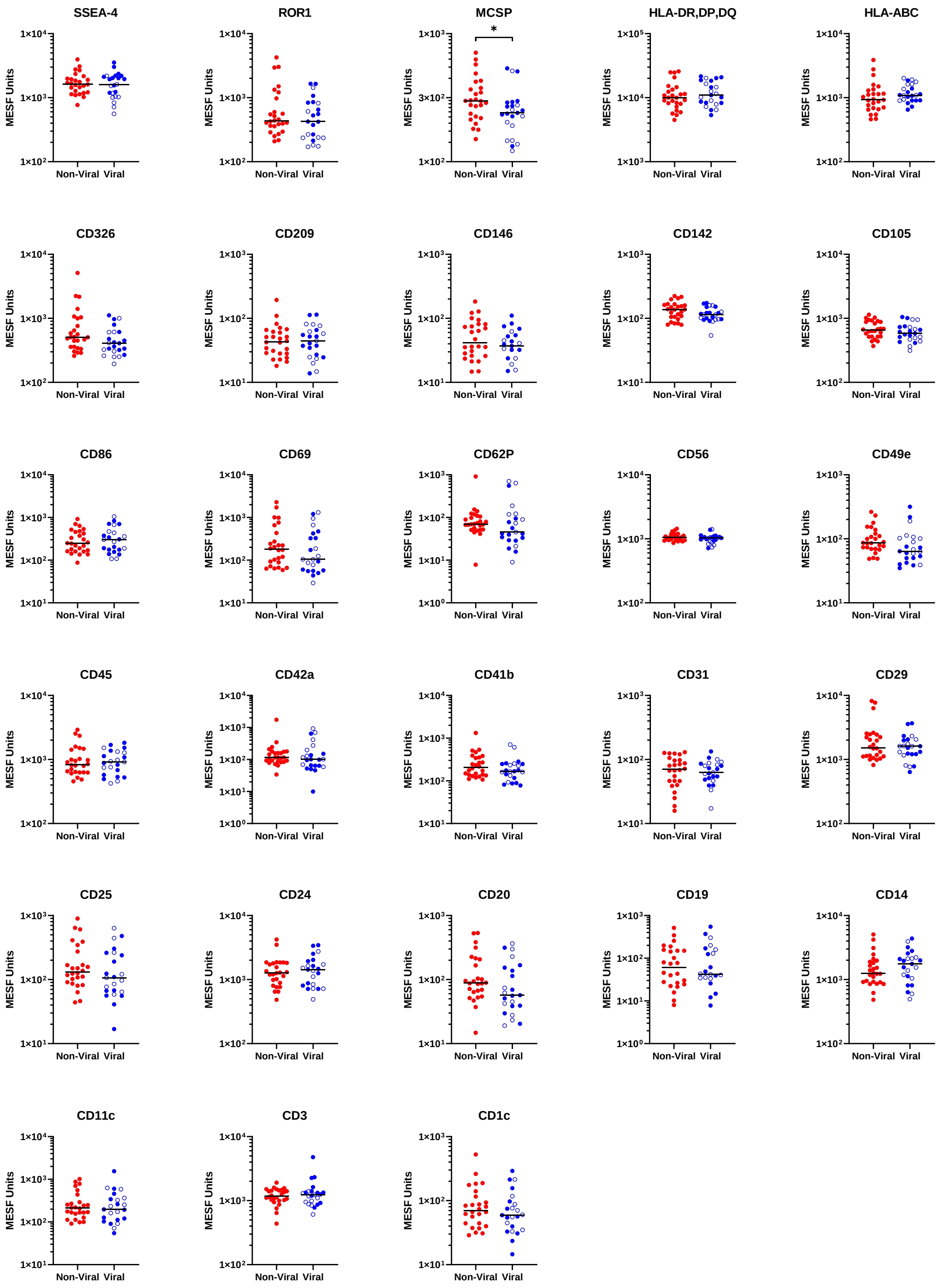

Supplemental Figure 6: MPA of additional CSF EVP surface markers compared by viral vs. non-viral disease etiologies. All additional MPA results of CSF EVPs from viral and non-viral disease etiologies are shown. Results were normalized and converted from .fcs file arbitrary unit scales to APC MESF units using reference standards and MPA_{PASS} software. Open blue circles represent data from HAM CSF EVPs. Nonparametric statistical analyses comparing groups were performed using Mann-Whitney tests for significance ($p \leq 0.05$ = *).

Supplemental Table 1: Patient cohort demographics from validation MPA. Healthy volunteers (HVs), HTLV-1-associated myelopathy (HAM), and multiple sclerosis (MS) patient demographics are shown by age in years and sex.

	# Patients	Male/Female	Median Age (Range)
**HV**	3	2/1	57 (42-75)
**HAM**	10	4/6	59 (45-75)
**MS**	17	1/9	51 (23-69)
